# Progress in Medicine

**Published:** 1899-12-23

**Authors:** 


### Progress in Medicine.

### RHEUMATISM AND GOUT.

(*Continued from page* 163.)

**Rheumatoid Arthritis.**—Bannatyne[21] met with a case where a patient suffering from typical arthritis deformans was attacked with diarrhœa, pericarditis, and pleurisy. Possibly true rheumatism may have co-existed, for there was a history of acute rheumatism in the past. Post-mortem destructive changes were found in the knee, and no out-growth or attempt at repair. He thinks that complications, such as mentioned above, do occur in true rheumatoid arthritis, though in this case he would assign them to a secondary infection, and he lays down that in acute forms of the disease no repair or enlargement of bones or cartilage takes place. The common over-growths are found either in chronic forms or in what is altogether a different disease. A very instructive discussion on the relation between this affection and gout took place at the North-West London Clinical Society. Ewart,[22] inquiring whether the two diseases ever co-exist or follow one another, lays down that an inherited gouty tendency seems not uncommon in sufferers from rheumatoid arthritis, and the inactive life the disease entails may tend to develop gout, while some who have suffered from gout may, from failure of nutrition, develop arthritis later on. Finally, some patients present a history of former gout and of rheumatic fever, and have well-marked Heberden's nodes.

Luff,[23] believing in the toxic theory of the origin of rheumatoid arthritis, thinks it may occasionally concur with or follow the injuries produced by gout, but that the two diseases are quite distinct and unrelated. Haig, on the other hand, regards both diseases and rheumatism as due to the same cause, and has seen the joints on one side of a body contain urates, and those on the other free from them, though on both sides the changes of rheumatoid arthritis were present. In debilitated persons urates are freely soluble in the blood, and keep up a constant irritation of the joints and tissues. If tonics and a full diet are given the blood is cleared of them, and the patient improved, as we see in rheumatoid arthritis. In acute febrile forms of these diseases salicylates indeed remove the uric acid in a harmless combination, but in chronic types it fails to cope with it, and we have therefore to fall back on acids, tonics, and full diet.

Savill[24] notices that in a large number of chronic joint cases, where tophi were absent, those which turned out to be gouty had generally the chief trouble confined to one joint; in rheumatoid arthritis almost all joints were affected, and in true chronic rheumatism the swelling was due to thickening of tissues outside the joints, and not to changes in the bones. For the rheumatoid cases no treatment equalled that of a pound of lean meat daily, with as much hot water as could be taken. In women he had found that the cure of catarrhal states of the uterus often led to a disappearance of chronic rheumatism or arthritis. Possibly the toxins were absorbed through mucous surfaces, whether in the tonsils or uterus. Jackson Clarke discusses the origin of a number of small deformities, such as hammer-toe and Dupuytren's contraction, and in children spinal trouble with affections of the finger-joints and swelling of the spleen, and, again, stiff joints following upon slight injuries. Some of these occur in persons showing symptoms of rheumatoid arthritis, and others in the subjects of gout. A man whose joints are irritable may be the subject of either disease, as his surroundings are favourable to one type or the other. Bannatyne[25] reviews the success of surgical treatment. In a few cases excision may be advisable for joints which are anchylosed and resist all ordinary treatment. Excision or scraping has been tried, not only to cure a given joint but to arrest the spread of the disease, and good results are reported in the cases where this has been done. In Collinson's patient it is noteworthy that the wasted muscles recovered after excision of the joint; and, as W. D. Parker mentions, the possibility of such recovery is confirmed experimentally, for the trophic changes in this disease can be delayed by section of the posterior roots of the spinal nerves.

**Gout.**—A. P. Luff[26] refers to the change of the sodium quadriurate into the gelatinous biurate, and then into the crystalline form, and points out that if we can delay the latter change time is given for elimination. He believes that in gout the kidneys fail to excrete the uric acid they form, and that much is absorbed from them into the general circulation leading to its deposit around the joints and their final irritation. He denies that the uric acid in solution has any irritating effects, and found that the alkalinity of the blood in a number of gouty patients was actually greater than in healthy persons. As this alkalinity is due to sodium salts the change into crystalline biurates is quicker than normal in such persons. Pot. bicarbonate has, however, been shown to delay the change. Hence, he recommends the use of colchicum, with citrate of potash and green vegetables. The joints themselves may be treated with massage, muscular exercise, cataphoresis, hot air, and other baths. Hopkins and Hope[27] have shown that the increased uric acid excretion which follows a meal can hardly be due to the nucleins ingested, but rather to some more soluble constituent, for the increase occurs before there is time for digestion of the nucleins. Some foods, too, cause increased leucocytosis without increased uric acid excretion, and others which contain hardly any nuclein cause great excretion. Critzman,[28] holding the renal theory of gout, remarks that the tubuli contorti are the first part of the kidney affected in interstitial nephritis, and it is just this part which is concerned with the excretion of uric acid. Thus, there is no gout without nephritis, though these tubules may be affected years before they show any direct clinical symptoms. Von Kossa,[29] of Buda Pesth, produced gout in birds by injecting oxalates, sugar, carbolic acid, acetone, or mercuric chloride. If inorganic substances such as chromates were used gouty deposits would be prevented by giving piperazin at the same time, but the latter has no effect if sugar or other organic substances are injected. The evidence of early kidney failure in gout is strengthened, according to S. A. Bontorl,[30] by the known reduced excretion of uric acid in plumbism where large quantities of the acid exist in the blood, while the kidneys correspond anatomically with those seen in early gout. Such kidneys, too, though they excrete urea, are unable to pass on the odoriferous bodies, and we thus find that in gout there is no odour of violets if turpentine is given. The kidneys, again, may be only affected at first in patches, and may be equal to their work except when a liver upset allows an unusual quantity of glycocine to reach them. This they combine with urea and so form uric acid in excess. Mouillot[31] urges that the gouty generally eat too much and drink too little. Vegetarian diet is not usually necessary, excessive muscular exertion should be avoided, and different types of food at the same meal, such as proteids and starches, are harmful to some patients. He maintains, too, that mineral waters containing sodium are of value. Indeed, Luff does not deny the use of soda in treating gouty affections of the liver, but merely its ability to dissolve uratic concretions, as an excess may hasten their deposition. Armstrong, in the discussion of this paper, said that with the beef and hot water treatment he found that there was an immensely increased excretion of uric acid at first, which ceased as the gouty troubles disappeared though the diet was unchanged.

O. Graham[32] praises massage and galvanism in gout, and mentions the successful treatment of a patient in a somewhat acute stage by general and then by local massage. The negative pole of a battery was afterwards applied to the swollen parts and followed by fresh massage. Very rapid improvement followed. He does not, however, appear to mention the danger of uræmic symptoms, which are sometimes seen after massage where the kidneys are crippled. Uricedin has been given in doses of from 30 to 90 grains daily by Holtz and others with some success. It should be stopped for a few days when the water becomes alkaline. It improves the digestion and appetite and relieves pains of gouty origin. Thialion is another remedy for lithæmic conditions recommended by E. C. Smith and Professor Goelet. It is given in teaspoonful doses in hot water and has an aperient action. Here too, if the water becomes alkaline, its use must be suspended. The Llangammarch waters are said by W. B. Jones and E. Russell[33] to increase both the amount of urine and also the uric acid excreted. The latter remains high for some time after the water is left off. Clinically, the value of the water is seen in the number of gouty patients at the spa who obtain benefit. Two Harrogate waters, the sulphur and chalybeate ones, seem, on the other hand, to reduce the amount of uric acid excreted; and Brain and Edgcombe, who determined these effects with precision,[34] note that tea and coffee have a similar effect, the amount rising rapidly when they are left off.

[21] Brit. Med. Jour., Oct. 11. [22] Inter. Med. Mag., April. [23] Clinical Jour., July 26. [24] Clinical Jour., Aug. 2. [25] Lancet, Nov. 18. [26] Lancet, Nov. 18. [27] Practitioner, p. 330, 1899. [28] Brit. Med. Jour., Mar. 11. [29] Archiv. Inter. de Pharmaco., vol. v. [30] Treatment, May 11. [31] Lancet, Mar. 23. [32] Lancet, July 1. [33] Lancet, Mar 25. [34] Med. Chron., Mar.

